# Supplementary material for: Immunotherapy Discontinuation in Metastatic Melanoma: Lessons from Real-Life Clinical Experience
Source: Cancers (Basel). 2021 Jun 20;13(12):3074. doi: 10.3390/cancers13123074 (PMC8234591; doi:10.3390/cancers13123074)
Supplement: Supplementary file 1 [file cancers-13-03074-s001.zip › cancers-1250738-supplementary.pdf]

cancers

MDPI

Supplementary Material: Immunotherapy Discontinuation in Metastatic Melanoma: Lessons from Real-Life Clinical Experience

Nethanel Asher, Noa Israeli-Weller, Ronnie Shapira-Frommer, Guy Ben-Betzalel, Jacob Schachter, Tomer Meirson and Gal Markel

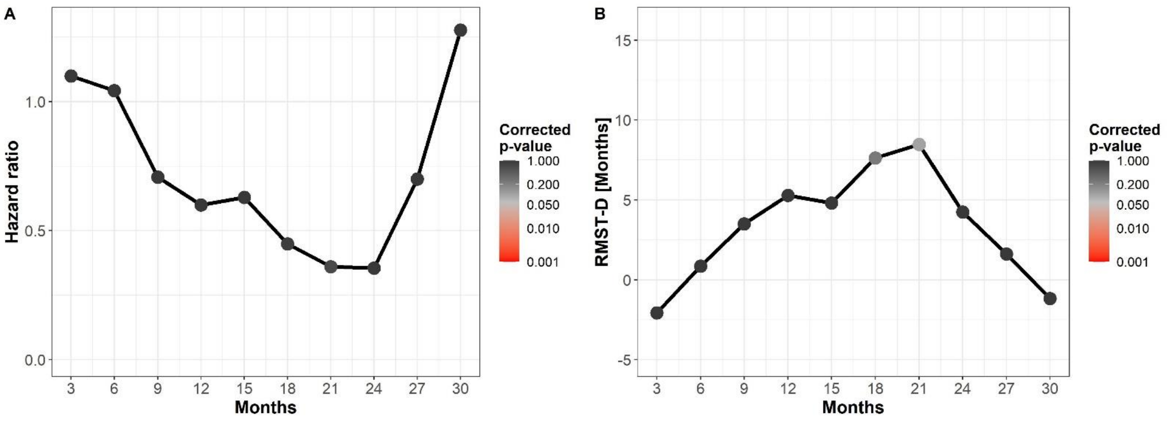

Figure S1. Influence of treatment duration on progression-free survival, calculated from treatment discontinuation. Shown are hazard ratio (HR) (left) and restricted mean survival time difference (RMST-D) (right) for progression-free survival (PFS) calculated from treatment discontinuation, of patients with complete response at 3-month time intervals. Each time point represents a duration cutoff that stratifies patients into short or long treatment duration based on the time of treatment discontinuation. The corrected p-values are shown in color and presented as columns at the top panels. As opposed to Figure 5, where PFS was calculated from treatment initiation, HR for PFS and RMST-D have no significant p-values at any timepoint.
